# Reversing stem cell aging

**DOI:** 10.18632/oncotarget.4403

**Published:** 2015-06-09

**Authors:** Jiyung Shin, Mary Mohrin, Danica Chen

**Affiliations:** Program in Metabolic Biology, Nutritional Sciences & Toxicology, University of California, Berkeley, CA, USA

The stem cell theory of aging postulates that aging is the result of the failure of tissue specific stem cells to replenish tissues and to sustain tissue function. Stem cell functions decline with age, consistent with the degeneration and dysfunction of aging regenerative tissues. Stem cell dysfunction is associated with premature aging and evidence is emerging to suggest that stem cell manipulation can extend lifespan [1]. Longevity factors tend to be enriched or activated in stem cells compared to their differentiated progeny and are essential for stem cell maintenance [2, 3], providing further support to the stem cell theory of aging.

Adult stem cells are maintained in a metabolically inactive quiescent state for prolonged periods of time. It was thought that stem cells stay quiescent as long as there is no physiological demand for proliferation. However, recent advancements in stem cell biology favor the view that stem cell quiescence is an evolved adaptation to reduce metabolic byproducts, preserve genomic integrity, and ensure stem cell maintenance [4].

Sirtuins regulate diverse cellular pathways that control stress resistance, metabolism, and aging. SIRT7, a mammalian sirtuin, is a chromatin binding protein that deacetylates H3K18 at specific gene promoters to repress transcription and to promote stress resistance [5-7]. SIRT7 is induced by mitochondrial protein folding stress and represses the expression of the mitochondrial translational machinery components via its interaction with nuclear respiratory factor 1 (NRF1) to alleviate stress and promote stress resistance [6]. This stress response is coupled to reduced mitochondrial activity and cell proliferation. This pathway is required for hematopoietic stem cells (HSCs) to sense mitochondrial protein folding stress, which is intrinsically associated with HSC transition from quiescence to proliferation, enabling HSCs to return to quiescence and prevent cell death [6].

**Figure 1:**
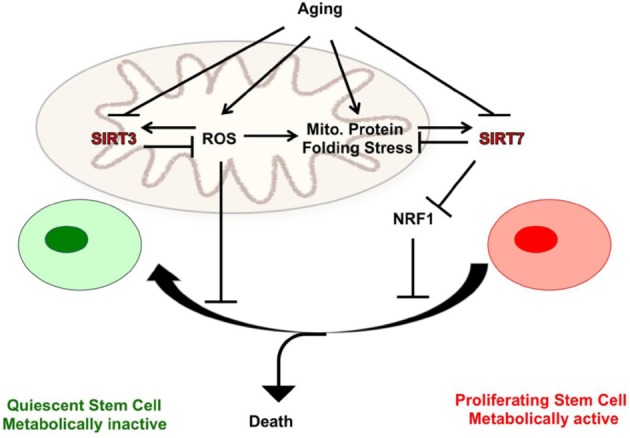
Reversing hematopoietic stem cell aging by SIRT3 and SIRT7 SIRT7 and SIRT3 converge at mitochondrial regulation to balance cell proliferation and cell survival. SIRT7 represses the NRF1 activity to alleviate mitochondrial protein folding stress, and to reduce mitochondrial activity and proliferation. SIRT3 reduces oxidative stress and promotes cell proliferation by modulating mitochondrial metabolic enzymes. These protective programs are repressed in aged hematopoietic stem cells and reintroduction of SIRT3 or SIRT7 improves the functional capacity of aged hematopoietic stem cells.

Mitochondrial protein folding stress emerges as an origin of cellular damage that causes HSC aging. The canonical mitochondrial unfolded protein response genes are upregulated and SIRT7 is repressed in aged HSCs [6]. Therefore, repression of the SIRT7-mediated protective program contributes to the elevated mitochondrial protein folding stress in aged HSCs. As another possibility, ROS have been shown to induce robust mitochondrial protein folding stress. ROS levels increase with age in HSCs and defective ROS management results in compromised HSC maintenance. It remains a possibility that high levels of ROS also contribute to elevated mitochondrial protein folding stress in aged HSCs and ROS-induced HSC defects are in part due to mitochondrial protein folding stress. It is interesting to note that SIRT3, a mitochondrial homolog of SIRT7, is highly enriched in HSCs where it regulates an oxidative stress response to reduce cellular ROS and is essential for HSC maintenance at an old age [2]. Therefore, SIRT3 and SIRT7 converge at mitochondrial protection to ensure HSC maintenance. These protective programs are repressed in aged hematopoietic stem cells and reintroduction of SIRT3 or SIRT7 improves the functional capacity of aged hematopoietic stem cells (Figure 1) [2, 6].

Thus, SIRT3 and SIRT7 may modulate the aging process by regulating stem cell quiescence and tissue maintenance. It will be of particular interest to establish whether other tissues use the same mechanism for maintaining stem cell quiescence. It will also be important to identify other genes that mediate mitochondrial protein folding stress to regulate stem cell quiescence. This knowledge will open new possibilities for regenerative medicine and treatment of diseases of aging.
